# Primary Small Cell Carcinoma of the Hypopharynx: A Report of Two Cases and Review of Nine Additional Cases

**DOI:** 10.1155/2017/8143145

**Published:** 2017-07-19

**Authors:** Mitsuhiko Nakahira, Kiyomi Kuba, Satoko Matsumura, Masashi Sugasawa

**Affiliations:** Department of Head and Neck Surgery, Saitama Medical University International Medical Center, 1397-1 Yamane, Hidaka, Saitama, Japan

## Abstract

**Objective:**

Two patients with primary small cell carcinoma (SmCC) of the hypopharynx, an extremely rare site for the occurrence of SmCC, are reported and nine additional well-documented cases are reviewed.

**Methods:**

Case report and review of the literature concerning primary SmCC of the hypopharynx.

**Results:**

On the final analysis, we reviewed eleven cases of primary SmCC of the hypopharynx. The tumors contained mixed elements of SmCC and squamous cell carcinoma (SCC) in six (55%) of eleven patients. Out of eleven patients, two patients had distant metastasis at the initial presentation. Even though nine patients presented with locoregional disease, development of distant metastasis after treatment was seen in five patients (56%), whereas there was no report of treatment failure on the primary site. To achieve more than two-year survival, patients should have received more than 4 cycles of chemotherapy.

**Conclusion:**

We report two cases of primary SmCC of the hypopharynx with a review of the literature. In more than half of the cases, combined carcinomas with SCC are seen. Because this tumor has a strong propensity for distant metastasis even in patients with clinically localized tumor, new powerful systemic agents should be explored.

## 1. Introduction

Small cell carcinoma (SmCC) is a common pulmonary neoplasm, which comprises approximately 10% of all pulmonary carcinomas in Japan [1]. It is increasingly recognized that SmCC may also arise in extrapulmonary sites throughout the body, commonly in the esophagus, large bowels, the bladder, the uterine cervix, and the larynx [2]. Similar to small cell lung carcinoma (SCLC), extrapulmonary SmCC (EPSmCC) is aggressive with rapid local progression and early regional and distant spread [2]. The incidence of EPSmCC is much lower than for SCLC accounting for only 6% of SmCCs [3, 4]. Most available literature on this condition exists in the form of case reports and retrospective series. The role of local and systemic therapies for EPSmCC is still not clearly defined [3, 4].

The first case of EPSmCC arising in the head and neck was a primary tumor in the larynx reported by Olofsson and Van Nostrand in 1972 [5]. Since then, it has been reported to occur in multiple sites of the head and neck throughout the upper aerodigestive tract, including the larynx, paranasal sinuses, and salivary glands [6]. However, in the head and neck, the hypopharynx is an unusual site for this tumor. Owing to the paucity of cases of primary SmCC of the hypopharynx, little information is available concerning their diagnosis and management. We report two cases of SmCC arising from the hypopharynx that occurred in a 75-year-old man and a 73-year-old man, along with a review of the available literature on primary SmCC of the hypopharynx.

## 2. Case Reports

### 2.1. Case  1

**Figure 1:**
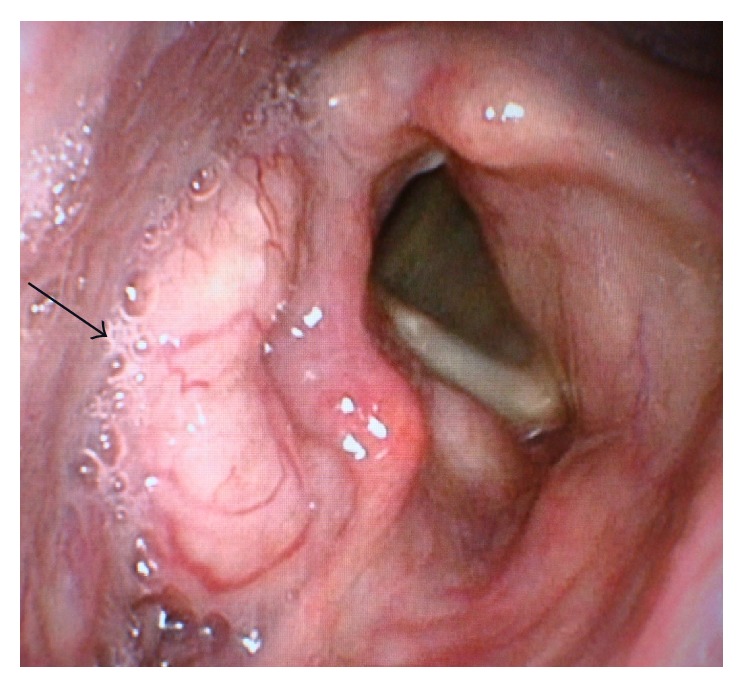
Fiberscopic view of the hypopharynx filled with a large soft tissue mass in the right pyriform sinus (arrow).

**Figure 2:**
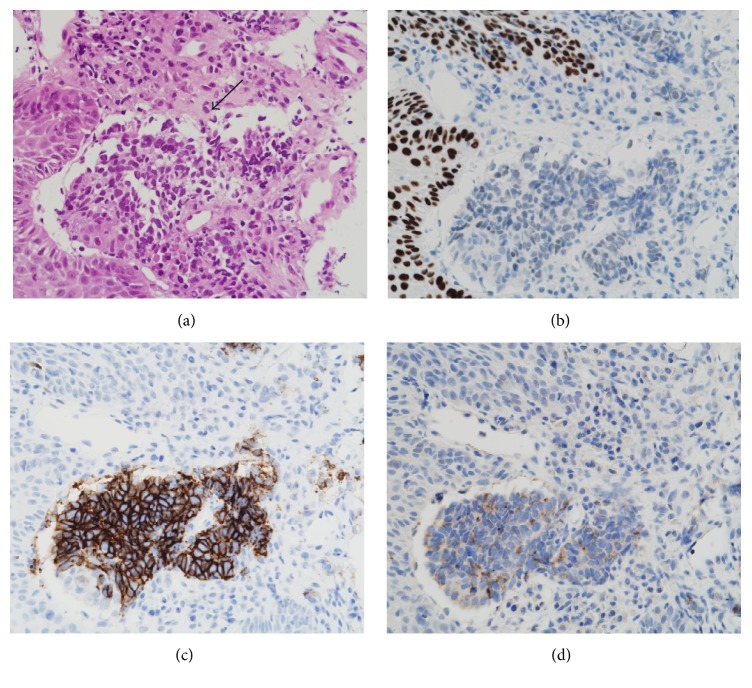
Histopathological and immunohistochemical features of the combined SmCC and SCC of the hypopharynx. (a) This tumor consists of a mixture of SmCC (center, arrow) and SCC (peripheral) (hematoxylin-eosin, original magnification 400x). (b) The p63 reactivity is seen in the nuclei of the cells of SCC but is not seen in SmCC component. (c) The CD56 reactivity in SmCC component is seen but is not seen in SCC component. (d) Chromogranin A shows positive cytoplasmic staining in SmCC component.

A 75-year-old man with 30 pack-years smoking history consulted his regular otolaryngologist because of a 3-month history of dysphagia. Laryngoscopy revealed a large tumor in the right pyriform sinus (Figure 1). A biopsy of the tumor showed features of neuroendocrine tumor suggesting SmCC and additional component of squamous cell carcinoma (SCC) (Figure 2). Fludeoxyglucose F 18 positron emission tomography (^18^F-FDG-PET) analysis disclosed multiple positive accumulations in vertebral bones besides the right pyriform sinus and bilateral neck lymph nodes. As a result, his tumor was the primary hypopharyngeal SmCC and classified as cT2N2cM1 (7th edition of the UICC and AJCC staging system). Chemotherapy was applied with 4 cycles of etoposide and cisplatin as a standard regimen usually intended for treatment of aggressive SCLC. The patient partially responded to the treatment but finally succumbed to the progression of the cancer six months after the treatment.

### 2.2. Case  2

**Figure 3:**
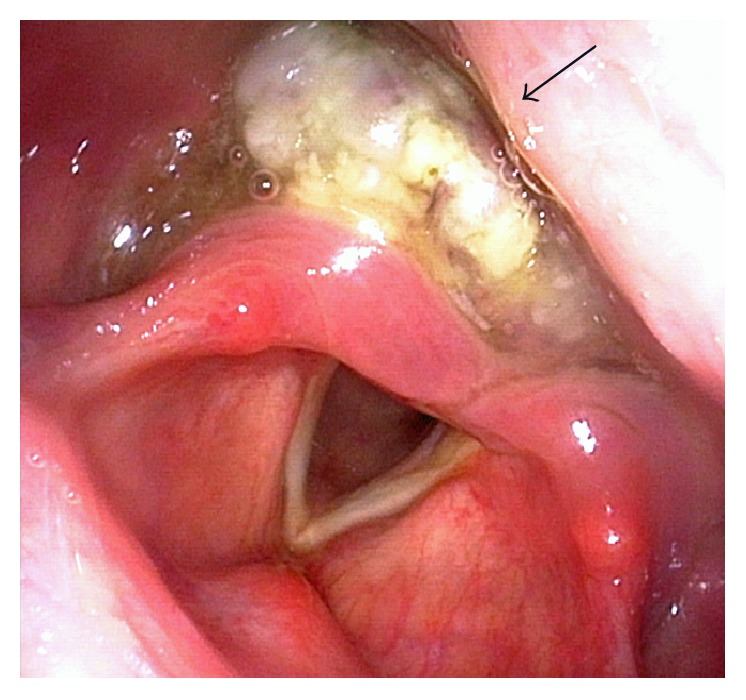
Fiberscopic view of the hypopharynx filled with a large soft tissue mass with necrotic appearance (arrow).

**Figure 4:**
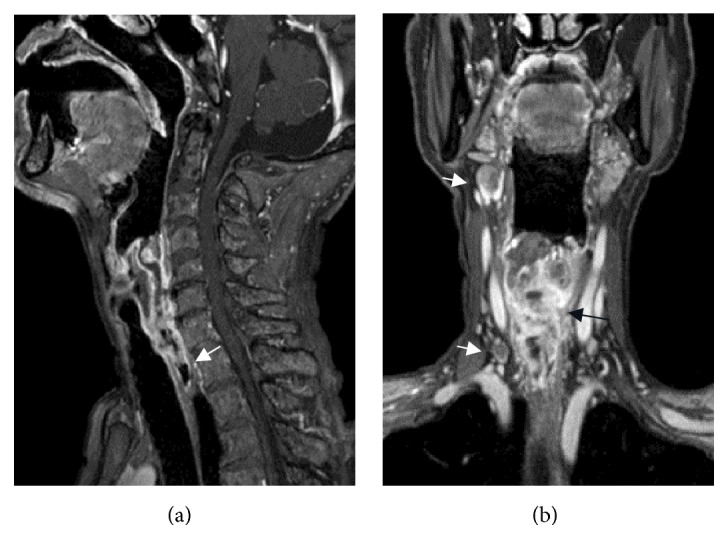
Preoperative cross-sectional MRI of the neck. (a) An enhanced sagittal image shows the tumor extending to the cervical esophagus (arrow). (b) An enhanced coronal image shows the large tumor completely occupying the hypopharyngeal space (black arrow). Note multiple lymph node metastases in the right neck (white arrows).

**Figure 5:**
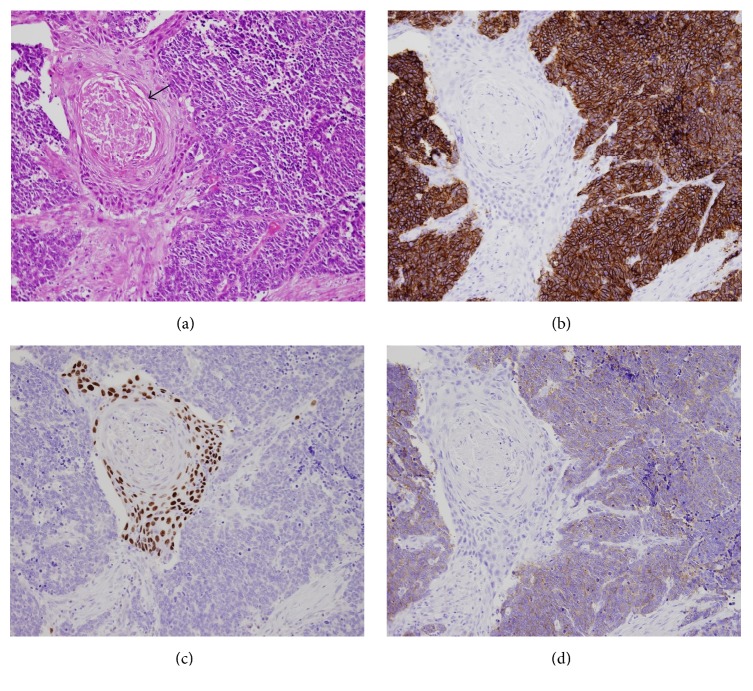
Histopathological and immunohistochemical features of the combined SmCC and SCC of the hypopharynx. (a) SmCC component coexisting with SCC component accompanying keratinization (arrow) (hematoxylin-eosin, original magnification 200x). (b) The CD56 reactivity in SmCC component is seen but is not seen in SCC component. (c) The p63 reactivity is seen in the nuclei of the cells of SCC but is not seen in SmCC component. (d) Synaptophysin shows positive cytoplasmic staining in SmCC component.

A 73-year-old man, with no history of smoking, presented with a two-month history of progressive dysphagia. Fiberscopic examination revealed a large mass arising from the hypopharynx with normal vocal cord movement (Figure 3). A biopsy of the tumor revealed that the tumor had features of neuroendocrine tumor suggesting pure SmCC. Magnetic resonance imaging (MRI) studies with intravenous contrast revealed that the tumor occupying the right pyriform sinus extended to the cervical esophagus with the right cervical lymph node metastases (Figure 4). ^18^F-FDG-PET analysis indicated a positive accumulation in the hypopharynx and the right neck and no evidence of pulmonary primary or distant metastases. On the basis of these clinical findings, the final diagnosis was made to be primary SmCC of the hypopharynx classified as cT3N2bM0 (7th edition of the UICC and AJCC staging system). As the tumor completely obstructed the hypopharynx, he was not able to eat liquid food or even receive a nasogastric tube placement. Because he had been suffering from malnutrition and being at risk for aspiration, he was considered as a high-risk candidate for chemotherapy or chemoradiotherapy. As he desired to resume eating as soon as possible at the sacrifice of his larynx, total pharyngolaryngectomy with jejunal autotransplant reconstruction was performed. On gross examination of the total pharyngolaryngectomy specimen, 5.5 cm × 2.6 cm × 4.0 cm mass located mainly in the right pyriform sinus was identified. The tumor extended inferiorly to involve the cervical esophagus. Histopathological examination showed the tumor was composed of two separate patterns which were SmCC lateralized to the pharyngeal side and SCC to the esophageal side. SCC deeply infiltrated beyond the underlying muscle layer, whereas SmCC showed relatively superficial stromal invasion without the muscle involvement. Both tumor components showed clear boundaries, but a focus of gradual transition was seen. In right neck, the metastatic tumor involved 2 out of 11 level II nodes, 2 out of 9 level III nodes, 1 out of 8 level IV nodes, and 1 out of 11 level VI nodes. Microscopic sections of the metastatic nodes also revealed combined SmCC and SCC. No metastatic lymph node was identified in the left neck. Immunohistochemical staining panel showed a diffuse positive reaction to synaptophysin and chromogranin A in SmCC component in both primary and metastatic sites. The cells from this component also showed positive reactions to CD56 and thyroid transcription factor 1 (TTF-1). The cells from the SCC component showed positive reactions to p63 and p40, while synaptophysin, chromogranin A, CD56, and TTF-1 were negative. The staining patterns of immunohistochemical study allow confirmation of combined small cell neuroendocrine differentiation and SCC in this tumor (Figure 5). After surgery, the patients received 4 cycles of systemic chemotherapy with carboplatin and etoposide at 4-week interval. Although the surgical margins were microscopically tumor-free, postoperative local radiation at a dose of 60 Gy and prophylactic brain irradiation at a dose of 25 Gy in 10 fractions were administered. At the 8-month follow-up, multiple pulmonary recurrence developed and the patient received salvage chemotherapy with 7-ethyl-10-[4-(1-piperidino)-1-piperidine] carbonyloxycamptothecin (CPT-11). However, Grade 3 CPT-11-related diarrhea at the first cycle resulted in the discontinuation of the therapy. Nevertheless, the patient was alive with disease for 26 months after the initial treatment.

## 3. Systematic Review of the Literature

We used the PubMed database for articles published up to May 2016 using the following relevant keywords: hypopharynx and (“small cell carcinoma” or “oat cell carcinoma” or “neuroendocrine carcinoma”), mixed with a hand search of reference sections of relevant papers, we found thirteen cases [7–18] of the primary hypopharyngeal origin since the first case was described in 1980 [7]. However, four cases [13–15, 17] were excluded from this study because they had no firm histopathological diagnosis for SmCC. On the final analysis, we reviewed nine well-documented cases of primary SmCC of the hypopharynx combined with the data from our patient. For each patient, information was collected on age at diagnosis, sex, smoking history, staging, presenting symptom, disease extent, subsites of the hypopharynx, histology, treatment modalities, and prognostic outcome (Table 1). On the basis of the analysis, out of 11 patients with SmCC of the hypopharynx, 7 patients had disseminated disease throughout the course.

## 4. Discussion

EPSmCC is a distinct rare entity accounting for 6% of all SmCCs [3, 4] and approximately 7 to 11% of cases involve the head and neck [3, 4]. SmCCs of the larynx are well investigated as to their clinical features because the larynx is the most common affected site in the head and neck and to date; there were over 160 reported cases [6]. On the contrary, the hypopharynx is an extremely rare site for the occurrence of SmCC as shown in this study. The present study showed that the clinical features of SmCCs were almost indistinguishable from SCC in this site, because presenting symptoms and clinical appearance are similar to SCC. The majority of those patients were males and presented after the age of 60 years with dysphagia and cervical mass. EPSmCC was reported as having a weak association with tobacco use [6]. All the tumors involved the pyriform sinus, where there is an area of predilection for SCCs. Therefore, a definitive diagnosis of SmCC should be made histopathologically. When specific structures of SmCC are histologically found in a tumor, particularly from an unusual location, such as the hypopharynx, routine immunohistochemical staining contributes to appropriate diagnosis and continuous early treatment as seen in the present report.

Interestingly, EPSmCC is associated with other histologic tumors like a SCC or an adenocarcinoma, referred to as combined carcinomas, because it might derive from a pluripotent stem cell that develops neuroendocrine features or a neoplastic stem cell with divergent differentiation potential [19]. The combined carcinomas are reportedly unusual representing less than 10% of SmCC of the larynx [20]. However, the present study showed that more than half of the patients with SmCC of the hypopharynx had tumors which contained mixed elements of SmCCs and SCCs. In case 2, as individual tumor components had specific vertical distribution, an improper diagnosis was made preoperatively based on small samples from fiberscopic biopsies as pure SCC. On the other hand, Uwa et al. [12] reported a case of combined SmCC in a patient with earlier diagnosis of SCC through biopsy. In addition, Aggarwal et al. [19] reported a case of combined SmCC of the larynx with SCC in which individual tumor components were lateralized on either side of the larynx. These examples raise the point that there is a potential risk of overlooking a component of SmCC in a case of nonsurgical treatment of the primary lesion, if a biopsy was carried out limited in a part of the SCC.

Retrospective studies such as case reports or single-institution case series form the only body of evidence available to help clinicians make difficult decisions concerning the treatment of this rare cancer, particularly site-specific local treatment. For the aforementioned reasons, SmCCs of the larynx are well reported for their treatment [2, 6, 21]. Because the prognosis of SmCC of the larynx is dismal, with 5-year survival rates of 5%, surgical resection such as total laryngectomy, which causes a severe decrease in quality of life, is not recommended, whereas combined chemoradiotherapy aiming at organ preservation is preferred [21]. In the present study, therapeutic approaches by which patients were treated varied widely among individuals, even if a patient had a limited disease at diagnosis. Regardless of therapeutic modalities for the primary lesion, local control was successfully achieved in all patients. Therefore, organ preservation strategy with platinum-based chemotherapy with concurrent radiotherapy for the primary lesion seems to be reasonable for SmCC of the hypopharynx as well. However, in contrast to SmCC of the larynx, there is a higher risk of failed completion of chemoradiotherapy in patients whose deglutition had been already severely compromised by advanced hypopharyngeal tumors such as our second patient. That population might be a candidate for radical surgery if possible instead of chemoradiotherapy.

The mainstay of treatment for EPSmCC is systemic chemotherapy, because these tumors are characterized by a high proclivity for metastatic dissemination even in patients with clinically localized tumor. As EPSmCC does not have a proven algorithm for treatment, it has generally been treated in a similar fashion to SCLC in terms of systemic chemotherapy [22]. The latest evidence-based clinical management guidelines for small cell lung carcinoma (National Comprehensive Cancer Network Guidelines for small cell lung carcinoma; http://www.nccn.org) recommend maximum 4–6 cycles of etoposide plus platinum agents such as cisplatin or carboplatin as adjuvant treatment for subjects of limited stage. In the present study, although 9 patients had clinically localized disease at diagnosis, 5 of 9 patients became disseminated status early after treatment. Out of 5 patients, 3 patients did not receive any chemotherapy and one patient underwent only two cycles of chemotherapy composed of carboplatin and etoposide. Less frequency of repetition of chemotherapy than recommended cycles (maximum 4–6 cycles as a standard) might lead to a failure after chemotherapy. In contrast, patients with locoregional lesions who received more than 4 cycles of chemotherapy could gain more than two-year survival, even if the late dissemination occurred. Although systemic chemotherapy could prolong survival, the present study showed that it provided very limited benefits. We need novel agents which can provide significant impact on patients' survival. Recent advances of immune checkpoint inhibitors are expected to produce a robust efficacy to patients' outcome in SmCC [23].

In terms of PCI for EPSmCCs, there is no prospective data because of the paucity of this disease. In the current study, only one patient underwent PCI and developed no brain metastasis, whereas other 10 patients had no brain metastasis without PCI. Recent publications [24, 25] concerning the utility of PCI for EPSmCCs advocate that the routine use of PCI in patients with this disease cannot be recommended, because of lower incidence of brain metastasis compared with that of SCLCs. Although rare, some patients with EPSmCC will develop brain metastasis. To clarify the role of PCI for EPSmCCs, a prospective multicenter research using an exhaustive gene expression analysis might be needed to find biological differences between patients with brain metastasis and those with long-term remission.

## 5. Conclusion

We report two cases of primary SmCC of the hypopharynx with review of the literature. Combined carcinomas with SCC are usually seen. Because primary SmCCs of the hypopharynx have a strong propensity for distant metastasis even in patients with clinically localized tumor, treatment with radical surgery alone may control the primary tumor but offer less chance for cure. Improvement of survival might be achieved by the emergence of new powerful systemic agents.

## Figures and Tables

**Table 1:** Review of the clinicopathological features of the reported 9 cases and our 2 cases of SmCC of the hypopharynx.

Number	Author	Year	Age/sex	Smoking	Stage (TNM)	Main symptom	Site	Extent of lesion at presentation	Histology	Locoregional TX	Adjuvant systemic TX	PCI	Metastases (observed throughout the course)	Follow-up
(1)	Ferlito and Polidoro	1980	57/M	+	T1N2bM0	Neck mass	PS	Locoregional	Combined with SCC	Radical S + RT	None	−	Cervical nodes, bone, soft tissue	Died of cancer 3.5 months after diagnosis
(2)	Mills et al.	1983	49/M	+	NA	Neck mass, dysphagia, weight loss	PS	Locoregional	Combined with SCC	Radical S + RT	None	−	Cervical nodes	Alive free of cancer 6 months after treatment
(3)	Baugh et al.	1985	63/F	−	T4a, more than N2c, M0	Dysphagia, cervical mass	PS	Locoregional	Pure SmCC	Incomplete S	Many times (more than four)	−	Cervical nodes	Alive free of cancer 55 months after diagnosis
(4)	Baugh et al.	1985	35/M	−	T3N2aM0	Dysphagia, weight loss	Base of tonsil to PS	Locoregional	Pure SmCC	RT	none	−	Cervical node	Alive free of cancer 21 months after diagnosis
(5)	Gaba et al.	2005	65/M	ex+	T4aN1M0	Dysphagia, weight loss	PS	Locoregional	Pure SmCC	Incomplete S + CRT	Many times (more than four)	−	Cervical node	Alive free of cancer more than 24 months after diagnosis
(6)	Sano et al.	2005	67/F	+	NA	Neck mass	PS	Locoregional	Pure SmCC	RT	Less than four	−	Cervical node, lung, liver	Died of cancer 13 months after diagnosis
(7)	Uwa et al.	2013	73/M	+	T4aN2bM0	Neck mass	PS	Locoregional	Combined with SCC	Radical S	None	−	Cervical and mediastinum nodes, liver, lung	Died of cancer 9 months after treatment
(8)	Bayram et al.	2015	50/M	+	T4aN2bM1 (lung)	Severe respiratory distress	PS	Locoregional-distant	Pure SmCC	Incomplete RT	Less than four	−	Cervical and lung	Alive free of cancer 15 months after treatment
(9)	Misawa et al.	2016	74/M	+	T2N0M0	Throat pain, hoarseness	PS	Local	Combined with SCC	CRT	None	−	Bone	Died of cancer 7 months after treatment
(10)	Nakahira et al.		75/M	+	T2N2cM1 (bone)	Dysphagia	PS	Locoregional-distant	Combined with SCC	None	Many times (more than four)	−	Bone, liver	Died of cancer 6 months after treatment
(11)	Nakahira et al.		73/M	−	T3N2bM0	Dysphagia	PS	Locoregional	Combined with SCC	Radical S + RT	Many times (more than four)	+	Cervical nodes, lung	Alive with cancer 26 months after treatment

TX: therapy, PCI: prophylactic cranial irradiation, NA: not available, PS: pyriform sinus, S: surgery, RT: radiation therapy, and CRT: concurrent chemoradiotherapy.

## References

[1] Sawabata N., Asamura H., Goya T. (2010). Japanese lung cancer registry study: first prospective enrollment of a large number of surgical and nonsurgical cases in 2002. *Journal of Thoracic Oncology*.

[2] Walenkamp A. M. E., Sonke G. S., Sleijfer D. T. (2009). Clinical and therapeutic aspects of extrapulmonary small cell carcinoma. *Cancer Treatment Reviews*.

[3] Wong Y. N. S., Jack R. H., Mak V., Henrik M., Davies E. A. (2009). The epidemiology and survival of extrapulmonary small cell carcinoma in South East England, 1970–2004. *BMC Cancer*.

[4] Grossman R. A., Pedroso F. E., Byrne M. M., Koniaris L. G., Misra S. (2011). Does surgery or radiation therapy impact survival for patients with extrapulmonary small cell cancers?. *Journal of Surgical Oncology*.

[5] Olofsson J., Van Nostrand A. W. P. (1972). Anaplastic small cell carcinoma of larynx: case report. *Annals of Otology, Rhinology & Laryngology*.

[6] Renner G. (2007). Small cell carcinoma of the head and neck: a review. *Seminars in Oncology*.

[7] Ferlito A., Polidoro F. (1980). Simultaneous primary oat cell carcinoma (Apudoma) and squamous cell carcinoma of the hypopharynx. *ORL*.

[8] Mills S. E., Cooper P. H., Garland T. A., Johns M. E. (1983). Small cell undifferentiated carcinoma of the larynx: report of two patients and review of 13 additional cases. *Cancer*.

[9] Baugh R. F., Wolf G. T., McClatchey K. D. (1986). Small cell carcinoma of the head and neck. *Head & Neck Surgery*.

[10] Gaba A., Mbaoma R., Breining D., Smith R. V., Beitler J. J., Haigentz M. (2005). Unusual sites of malignancies: case 1. Small-cell carcinoma of the hypopharynx. *Journal of Clinical Oncology*.

[11] Sano M., Kitahara N., Toma M. (2005). Hypopharyngeal small cell carcinoma: a case report. *Auris Nasus Larynx*.

[12] Uwa N., Terada T., Mohri T. (2013). Combined small cell carcinoma of the hypopharynx. *Auris Nasus Larynx*.

[13] Takagawa R., Tanaka K., Yamada M. (2011). Primary neuroendocrine carcinoma of the hypopharynx: a case report. *Diseases of the Esophagus*.

[14] Milroy C. M., Robinson P. J., Grant H. R. (1989). Primary composite squamous cell carcinoma and large cell neuroendocrine carcinoma of the hypopharynx. *The Journal of Laryngology & Otology*.

[15] Kusafuka K., Abe M., Iida Y. (2012). Mucosal large cell neuroendocrine carcinoma of the head and neck regions in Japanese patients: a distinct clinicopathological entity. *Journal of Clinical Pathology*.

[16] Bayram A., Akay E., Göksu S. S., Özcan İ. (2015). Primary small cell carcinoma of the hypopharynx: a case report of a rare tumor. *Case Reports in Otolaryngology*.

[17] Lee W.-I., Ameratunga M., Du Plessis J., Gan H. (2015). Hypopharyngeal large cell neuroendocrine carcinoma. *BMJ Case Reports*.

[18] Misawa K., Kawasaki H., Endo S. (2016). Primary combined small and squamous cell carcinoma of the hypopharynx: a case report. *Molecular and Clinical Oncology*.

[19] Aggarwal G., Jackson L., Sharma S. (2010). Primary combined small cell carcinoma of larynx with lateralized histologic components and corresponding side-specific neck nodal metastasis: report of a unique case and review of literature. *International Journal of Clinical and Experimental Pathology*.

[20] Bares L., Bares L., Eveson J. W., Reichart P., Sidransky D. (2005). Neuroendocrine tumors. *World Health Organization Classification of Tumors, Pathology and Genetics of Head and Neck Tumors*.

[21] Ferlito A., Silver C. E., Bradford C. R., Rinaldo A. (2009). Neuroendocrine neoplasms of the larynx: an overview. *Head and Neck*.

[22] Brennan S. M., Gregory D. L., Stillie A., Herschtal A., Manus M. M., Ball D. L. (2010). Should extrapulmonary small cell cancer be managed like small cell lung cancer?. *Cancer*.

[23] Paglialunga L., Salih Z., Ricciuti B., Califano R. (2016). Immune checkpoint blockade in small cell lung cancer: is there a light at the end of the tunnel?. *ESMO Open*.

[24] Naidoo J., Teo M. Y., Deady S., Comber H., Calvert P. (2013). Should patients with extrapulmonary small-cell carcinoma receive prophylactic cranial irradiation?. *Journal of Thoracic Oncology*.

[25] Coca-Pelaz A., Devaney K. O., Rodrigo J. P. (2016). Should patients with laryngeal small cell neuroendocrine carcinoma receive prophylactic cranial irradiation?. *European Archives of Oto-Rhino-Laryngology*.

